# Exceptional Elasticity of Microscale Constrained MoS_2_ Domes

**DOI:** 10.1021/acsami.1c13293

**Published:** 2021-10-01

**Authors:** Cinzia Di Giorgio, Elena Blundo, Giorgio Pettinari, Marco Felici, Antonio Polimeni, Fabrizio Bobba

**Affiliations:** † Department of Physics E.R. Caianiello, University of Salerno, 84084 Fisciano, Italy; ‡ INFN, Sezione di Napoli, Gruppo Collegato di Salerno, Complesso Universitario di Monte S. Angelo, 80126 Napoli, Italy; § Physics Department, Sapienza University of Rome, 00185 Rome, Italy; ∥ Institute for Photonics and Nanotechnologies (CNR-IFN), National Research Council, 00156 Rome, Italy; ⊥ CNR-SPIN, 84084 Fisciano, SA, Italy

**Keywords:** elasticity, two-dimensional materials, bulged membranes, nanoindentation, force−distance curves, adhesion energy

## Abstract

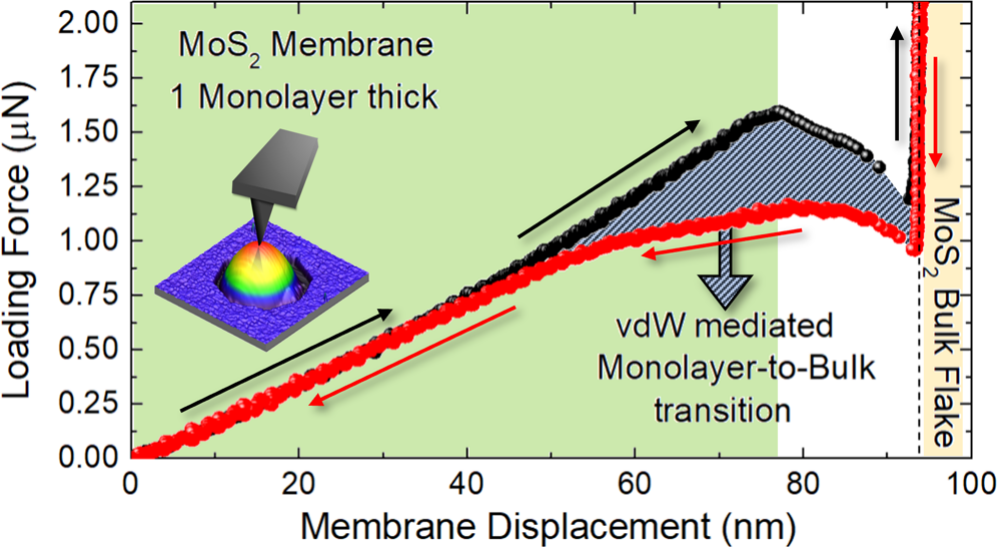

The outstanding mechanical performances of two-dimensional (2D) materials make them appealing for the emerging fields of flextronics and straintronics. However, their manufacturing and integration in 2D crystal-based devices rely on a thorough knowledge of their hardness, elasticity, and interface mechanics. Here, we investigate the elasticity of highly strained monolayer-thick MoS_2_ membranes, in the shape of micrometer-sized domes, by atomic force microscopy (AFM)-based nanoindentation experiments. A dome’s crushing procedure is performed to induce a local re-adhesion of the dome’s membrane to the bulk substrate under the AFM tip’s load. It is worth noting that no breakage, damage, or variation in size and shape are recorded in 95% of the crushed domes upon unloading. Furthermore, such a procedure paves the way to address quantitatively the extent of the van der Waals interlayer interaction and adhesion of MoS_2_ by studying pull-in instabilities and hysteresis of the loading–unloading cycles. The fundamental role and advantage of using a superimposed dome’s constraint are also discussed.

## Introduction

Two-dimensional (2D) crystals, with monoatomic or ultrathin structure, are a class of materials with emerging and promising properties. Akin to graphene, they are characterized by high in-plane stiffness and low flexural rigidity,^1^ so that individual atomic sheets are intrinsically capable of sustaining much larger mechanical strains compared to conventional semiconductors.^2^ In addition, mechanical strain can strongly perturb the 2D material’s band structure, giving rise to the possibility of using mechanical deformations to change the electronic and photonic properties and to tune the performances of 2D material-based devices.^3−6^ The exploration of coupling between mechanics and other physical properties, such as the thermal, electronic, and optical ones, is thus of fundamental relevance for novel applications. Recent experimental achievements in the application of mechanical strain to 2D materials^6^ mostly rely on substrate-supported setups. Commonly used strategies to induce in-plane deformations are indeed based on (i) epitaxial growth of 2D materials with controlled lattice mismatch;^7,8^ (ii) thermally driven lattice mismatch;^9,10^ (iii) the use of flexible substrates to easily stretch, compress, and/or bend the upper lying membranes;^11,12^ and (iv) the use of piezoelectric substrates.^13,14^ On the other hand, out-of-plane deformations can be caused by (i) wrinkles or buckle delamination, occurring because of compression;^15,16^ (ii) trapping of water or gas at the interface between the 2D crystal and its substrate, with the consequent formation of blisters (bubbles or tents);^17−19^ (iii) transfer of 2D layers on top of patterned or nanoparticle-engineered substrates;^20,21^ and (iv) bulging or poking the 2D crystal using microcavity-based setups,^22,23^ plasma treatments,^24,25^ and nanoindentation.^2,26,27^ In this broad scenario, the blister or bulged configurations have become an exceptional platform to test and measure all of the relevant mechanoelastic properties of 2D materials. Raman spectroscopy, as well as atomic force microscopy and spectroscopy (AFM/S), have indeed been employed to investigate mechanical deformation and layer delamination and to measure the relevant parameters, such as stress/strain, 2D Young’s modulus, and adhesion energy.^17,23,28,29^ Concomitantly, other techniques such as photoluminescence and scanning tunneling microscopy (STM) were used to study the correlation between the applied strain and tunability of the electronic and optoelectronic properties.^24,30,31^

Compared to the other techniques, AFM/S has the advantage of providing accurate measurements of interface forces (with a resolution of the order of pN), whose impact on the performances of micro- and nanosystems is to date at the forefront of physics and materials science. In addition, AFM/S is capable of providing local measurements of material properties such as elasticity, hardness, and adhesion, with nanometric resolution. In particular, the van der Waals (vdW) force-driven adhesion phenomenon plays a prominent role in 2D materials. Indeed, while the crystal structure and properties of widely used metals and semiconductors are governed by the setting up of covalent bonding, 2D materials exhibit strong covalent in-plane bonds accompanied by a weak vdW out-of-plane interaction, which allows for the relatively easy exfoliation of monolayers or few layers.^32^ Finally, vdW forces also play a pivotal role in the fabrication and engineering of 2D heterostructures.^33^

In the present paper, we will use AFM/S-based nanoindentation to investigate (i) the elasticity and robustness of tensile-strained membranes, herein called domes, produced with site control in bulk molybdenum disulfide (MoS_2_) and (ii) the interlayer MoS_2_ adhesion. Among the 2D family of semiconducting transition metal dichalcogenides (TMDs), MoS_2_ is one of the most promising members, given the high tunability of its electronic and optoelectronic properties on the hydrostatic pressure,^34,35^ number of layers,^36−38^ local strain,^30,39−42^ and interfaced materials.^43,44^ The strength of our approach relies on the capability of producing monolayer-thick MoS_2_ domes by inducing local delamination directly from the bulk MoS_2_ substrate by H-ion irradiation,^24^ thus avoiding layer transferring and complex substrate preparation. Such domes are characterized by an anisotropic tensile in-plane strain that increases from the edge toward the summit, where it becomes isotropic-biaxial.^24,42^ In addition, constrained, equally sized and spaced bubbles can be produced by exploiting the fabrication approach, based on the realization of lithographically defined H-opaque mask, first proposed in ref (24). Furthermore, this approach allows us to further increase the built-in strain of the domes, achieving biaxial strains as high as 7–8%.^28,29^ The as-fabricated domes, loaded by the local AFM nanoindentation, showed exceptionally high robustness upon the herein called *crushing procedure*. Such a procedure implies a loading force of the order of μN and indentation depth as large as the dome’s height. Moreover, the full reversibility of the process makes MoS_2_ domes the pristine platforms for evaluating the mechanical behavior of 2D membranes under stress and to derive their elastic properties, beyond their interest in fundamental physics and materials science research. Furthermore, we report on pull-in instabilities of the loading curves, addressable to the vdW interaction between the indented membrane and the bulk substrate. This result, together with the analysis of the loading–unloading cycle, provides an innovative method to quantify relevant physical parameters, such as the inner dome pressure and the MoS_2_–MoS_2_ adhesion energy. Finally, we highlight the real advantage of fabricating constrained bubbles for the evaluation of their elasticity, by investigating the behavior of unconstrained blisters and their slippage under AFM loading.

## Results and Discussion

We performed AFM imaging and nanoindentation of equally sized domes produced in MoS_2_ bulk flakes via hydrogen (H)-ion irradiation. As described elsewhere,^28,29^ MoS_2_ flakes are first mechanically exfoliated onto a Si/SiO_2_ substrate and, subsequently, partially coated by a hydrogen silesquioxane (HSQ) H-opaque layer. Octagonal openings of micrometer-scale radius are then produced in the HSQ layer via electron-beam lithography (EBL) and, finally, low-energy H-ion irradiation is performed on the whole sample surface. By doing so, localized protrusions appear on the flake surface on the uncovered area and within the octagonal openings, in the shape of domes, due to the accumulation of hydrogen molecules in the crystal interlayer region. Thanks to the EBL-defined mask, regular arrays of domes with uniform, arbitrary size distribution can be achieved in the patterned flakes. In contrast, uncoated MoS_2_ flakes reveal the formation of randomly distributed domes of random size.^28,29^

**Figure 1:**
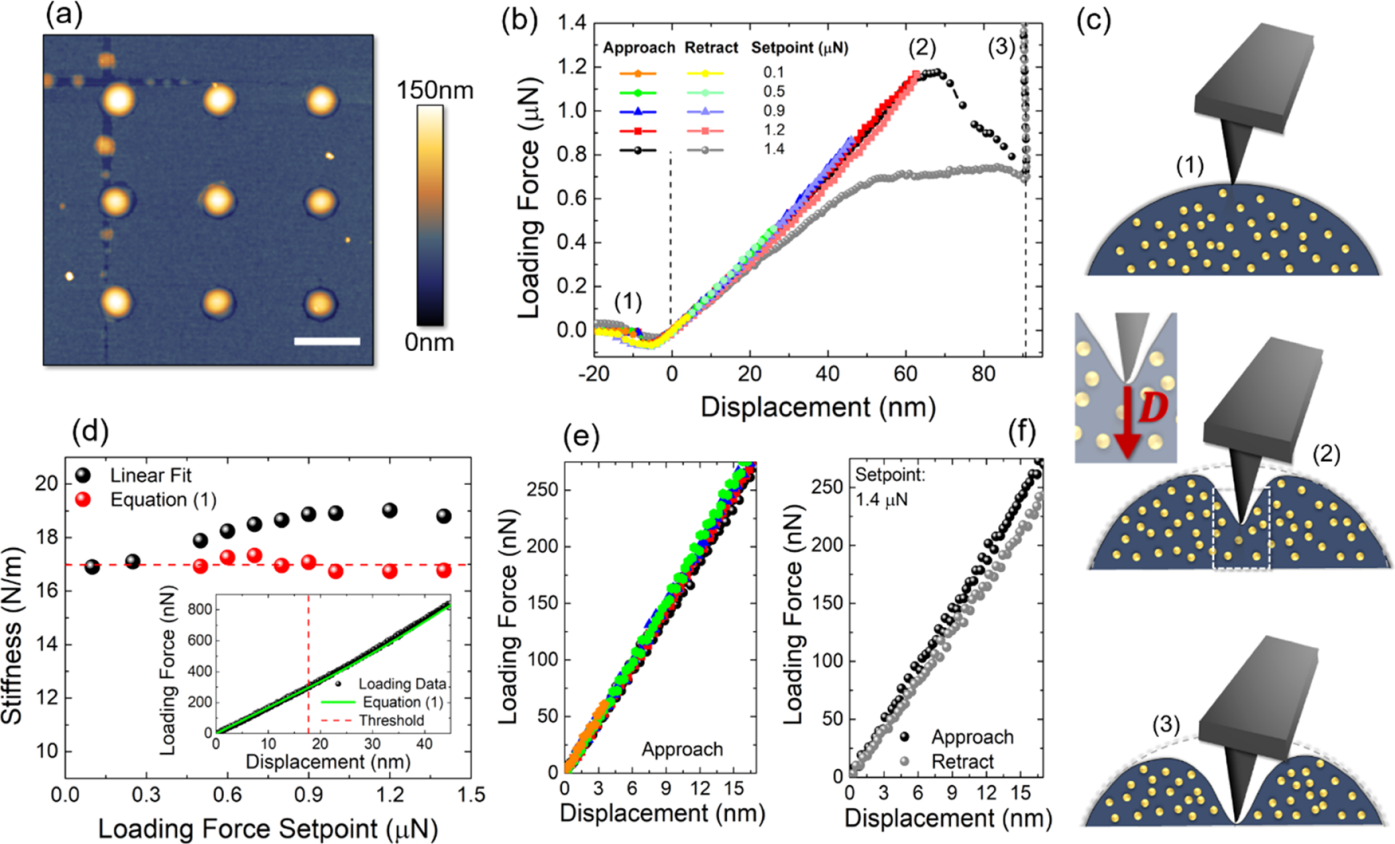
(a) Tapping-mode AFM topography, 10 μm × 10 μm in lateral size, of the HSQ-coated MoS_2_ surface after hydrogenation; scale bar: 2 μm. (b) Approach and retract FDCs of multiple indentations performed on the same dome by gradually increasing the force setpoint. (c) Scheme depicting the membrane arrangement under AFM loading in (1), (2) and (3) of (b). (d) Main panel: stiffness vs loading force setpoint measured using a linear fit (black scatters) and using eq 1 (red scatters); inset: typical FDC (black scatters), fitted by eq 1 (green line). The red dashed line indicates the threshold between linear and nonlinear regimes. (e) Close-up of the approach FDCs in the small indentation range. (f) Close-up of the approach and retract FDCs, measured using a force setpoint of 1.4 μN, in the small indentation range.

Figure 1a shows a typical tapping-mode AFM topography, 10 μm × 10 μm in lateral size, of the hydrogenated HSQ-coated MoS_2_ surface, revealing the nucleation of an array of almost equally sized and equally spaced H_2_-bulged domes, protruding from ∼1 μm wide openings in the 30–50 nm-thick HSQ layer. We performed nanoindentation by moving the AFM probe to the summit of a dome and acquired the loading force vs displacement curve (FDC), for a given loading force setpoint (maximum force exerted by the probe during the indentation). Figure 1b shows FDCs of multiple indentations performed on the same dome by increasing, time by time, the preset force (setpoint). Both approach and retract FDCs are plotted per cycle, obtained by pushing the AFM probe against the dome first (approach or loading) and pulling it away afterward (retract or unloading). A total of 10 cycles were acquired by increasing the preset force from a minimum setpoint of 100 nN to a maximum of 1.4 μN (only 5 cycles were included in Figure 1b for clarity of readability). Up to a 900 nN setpoint, approach and retract FDCs are not fully distinguishable in the plot since they overlap each other. A small hysteresis opens between the approach and retract FDCs acquired with a 1.2 μN setpoint (red and light-red scatters, respectively). Such a hysteresis gets much bigger when increasing the setpoint to 1.4 μN (black and gray scatters). In this case, the approach FDC shows three remarkable features: (1) snap-to-contact at the tip–dome contact point; (2) pull-in instability with a decrease of the force, at ∼70 nm indentation; (3) stiff increase of the force (vertical line) when reaching the bulk MoS_2_ substrate (the distance between (1) and (3) is compatible with the dome’s height). The top, middle, and bottom panels of Figure 1c depict schematically the membrane arrangement under the tip loading of each step: (1), (2), and (3), respectively. While the details of such features will be further discussed later in the text, we will now focus on the first region of the FDC (indentation ≤ 70 nm). A typical FDC for indentation against a pressurized object is expected to undergo a transition from linear (*F* = *k*δ) to cubic (*F* = αδ^3^) behavior, depending on the indentation range, small and large, respectively, with δ being the displacement caused by the loading force *F*. No asymptotic results are known to be able to transit smoothly between the two ranges so that the intermediate regime is commonly fitted by summing the results of small and large indentation limits, even though the outcoming error was demonstrated to be very large and dependent on the indenter’s size.^45^ As a matter of fact, the cubic regime is never reached in the presented experiments and to avoid unwanted fit inaccuracies, we implemented the fitting approach developed in ref (28). We considered a combination of linear (*F*_L_ = *k*δ) and nonlinear (*F*_NL_ = αδ^ω^) components, both weighted by the Heaviside function Θ(δ – δ_T_), with δ_T_ being the depth threshold between the linear and nonlinear regime. The stiffness *k*, the parameter α, the exponent ω, and the threshold δ_T_ are found by the optimization of the fitting procedure
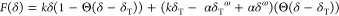
1

The inset of Figure 1d shows a good agreement between the experimental data (black scatters) and the fitting model (green line), highlighting with a red dashed line the threshold between the linear and nonlinear regime, the latter having ω = 1.3. The power dependence of *F*(δ), achieved in the intermediate regime of the present experiments, is small but not negligible in the measurement of the dome’s mechanical properties (such as the stiffness *k*, ultimately related to the internal pressure and 2D Young’s modulus^45^) unless incurring in inaccurate results. To detail the importance of including the nonlinear component in the FDC fit, the main panel of Figure 1d shows how the stiffness changes as a function of the loading force setpoint, depending on the used fitting model. Indeed, when fitting the whole FDC (in the range δ ≤ 70 nm) using only a linear component, the values of stiffness (black scatters) oddly depend on the loading force setpoint used. On the contrary, those values collapse on a straight horizontal line (17.0 ± 0.2 N/m) by implementing the model described by eq 1 (red scatters). It is worth mentioning that when performing multiple indentations on the same dome, we do expect the stiffness to be independent of the loading setpoint (unless irreversible changes are induced in the object as a consequence of indentation), making the results of the first method (linear fit over the whole indentation range) inaccurate. In addition, our model suggests a transition from linear to nonlinear behavior, occurring at 17.1 ± 0.3 nm indentation, so that the FDCs acquired with preset forces of 100 and 250 nN (4 and 12 nm indentation, respectively) only disclose a linear behavior. Figure 1e is a close-up of the approach FDCs in the small indentation regime (indentation: ≤ 17.1 nm), showing that they almost all overlap each other, thus confirming that they all have the same slope or the stiffness *k* = 17.0 ± 0.2 N/m. Finally, in Figure 1f, we plotted both the approach and retract FDC for a 1.4 μN setpoint, in the range of small indentation. One can notice that even if an abrupt change in the dome’s mechanical response is measured during the approach when the loading force overcomes 1.2 μN (black curve in Figure 1b), the retract curve (grey scatters), obtained when pulling the AFM-probe away, nearly perfectly overlaps back with the approach one (black scatters) upon retracting to small indentations (<6 nm). This result proves that the dome can withstand very large loading and deformation without permanent damage, ultimately suggesting an analogy with *superelastic* materials.^46^ While the details of this analogy are discussed in Supporting Information 1, here we stress the peculiarities of the measured FDCs. The latter, indeed, disclose (i) two subsequent elastic branches, representative of indentation on the bulged MoS_2_ monolayers, (1) and (2), and bulk, (3), respectively; (ii) a large hysteresis when performing a loading–unloading cycle because of the system transition from monolayer to bulk; and (iii) full reversibility of the whole process. The coexistence of these unique elastic properties corroborates the analogy between the elastic behavior of constrained MoS_2_ domes and conventional superelasticity.^46^ We attribute the setting up of such a superelastic-like behavior to the combination of (i) extreme MoS_2_ monolayer strength, allowing for fully reversible S–Mo–S inplane bond stretching;^47^ and (ii) a shape-recovery mechanism due to the competition between the van der Waals (vdW) attraction (which would favor the adhesion between topmost membrane and bulk) and the H_2_ gas action (which would restore the initial dome shape).

It is worth stressing that we chose to use AFM cantilevers whose elastic properties would have been suitable for a precise estimate of the dome’s stiffness, rather than that of the bulk. The latter is indeed much harder, thus giving rise to an immediate almost vertical response to the AFM loading (Supporting Information 2).

**Figure 2:**
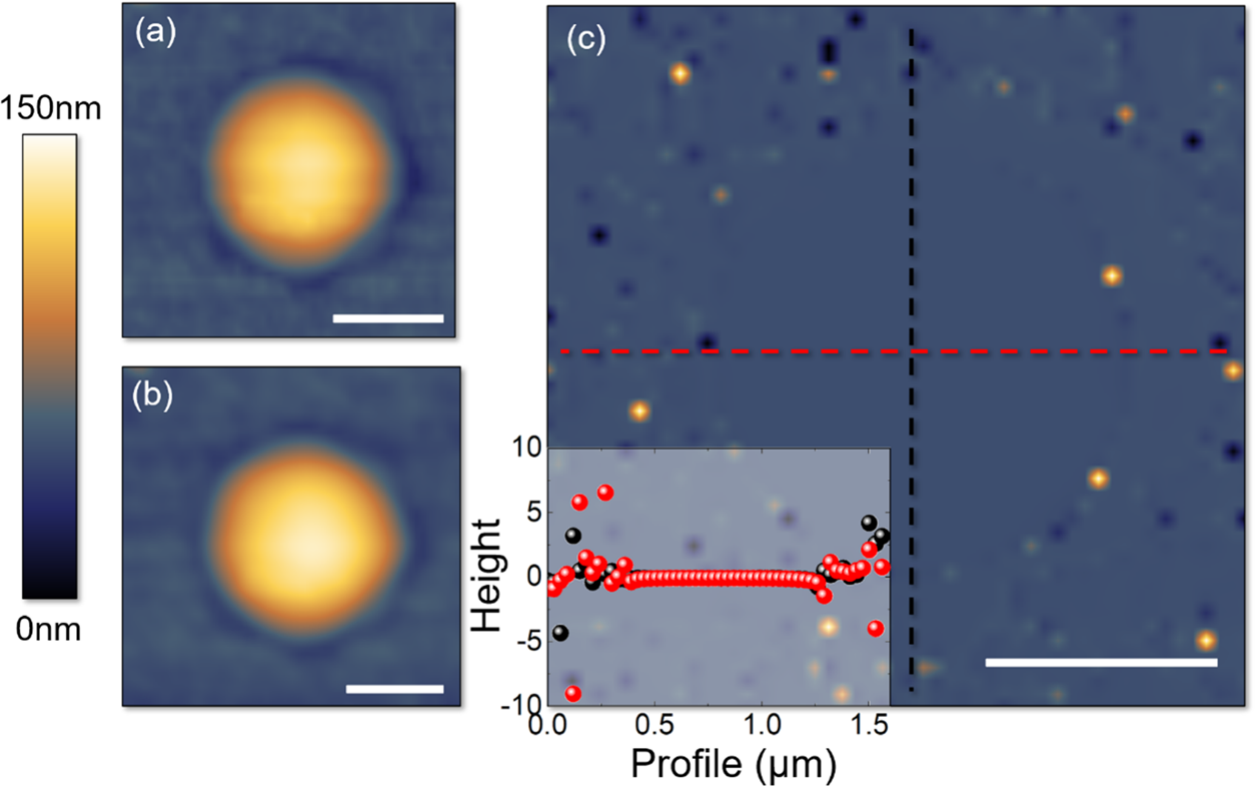
(a) Tapping-mode AFM topography, 1.6 μm × 1.6 μm in lateral size, of the same dome (a) before and (b) after the multiple indentation procedure. (c) Main: point-by-point normalized difference map *N*. Inset: profiles of the normalized height along the two orthogonal black and red directions in (c). Scale bar: 0.5 μm.

The dome’s robustness is also confirmed by comparing AFM images before and after the crushing procedure. Figure 2a,b shows the tapping-mode AFM topography, 1.6 μm × 1.6 μm in lateral size, prior to performing the nanoindentation cycles (*P*) and when the process is over (*O*), respectively. To check whether the dome has undergone any change in shape and/or size, the point-by-point normalized difference map  was calculated and is shown in Figure 2c, together with two profiles, evaluated along the black and red orthogonal directions. *N* is zero at every point inside the dome’s area, thus confirming that its shape and size are unchanged by the indentation procedure.

**Figure 3:**
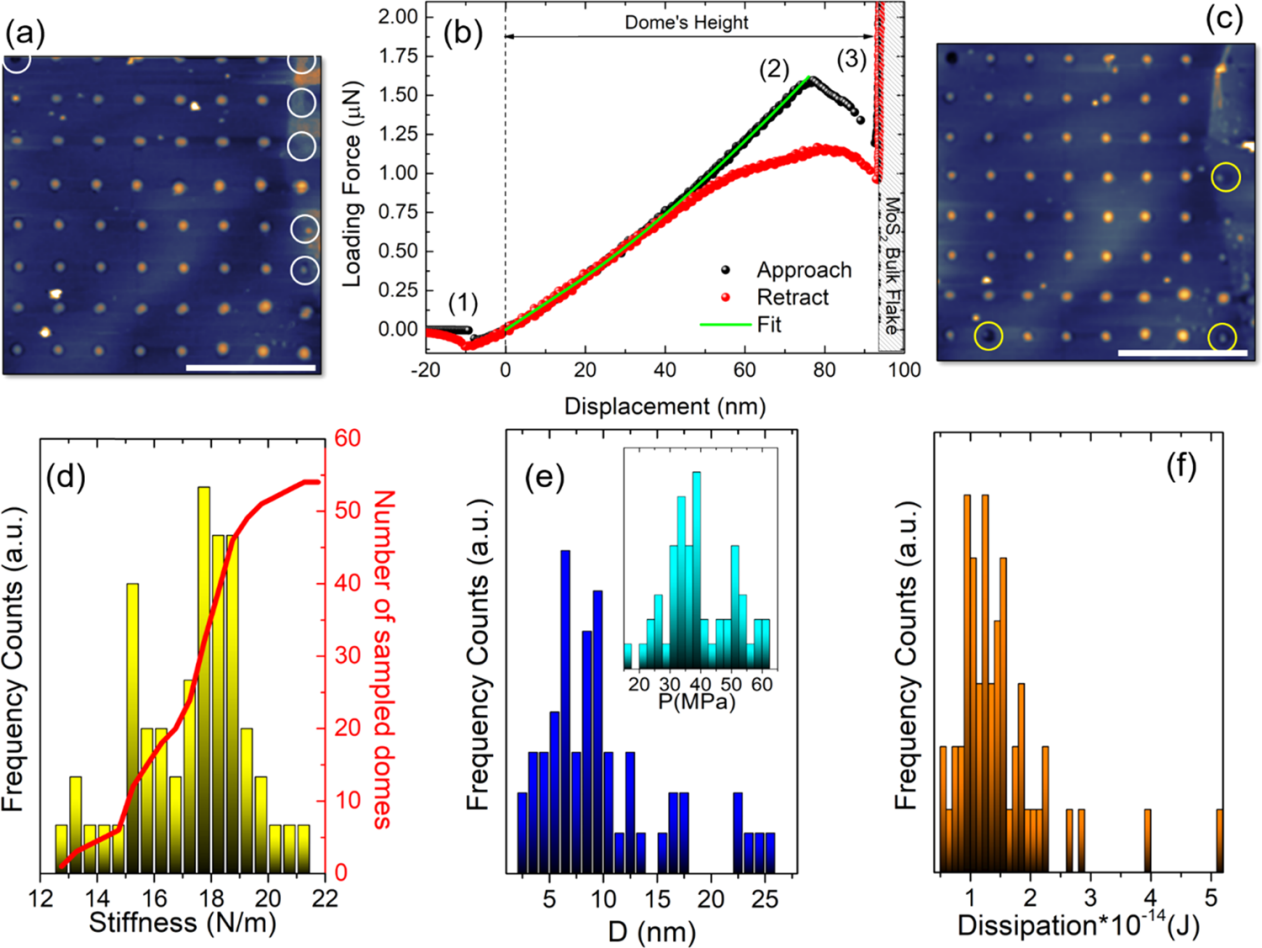
Tapping-mode AFM topography, 24.5 μm × 24.5 μm in lateral size, of an 8 × 8 opening’s array before (a) and after (c) performing the nanoindentations; scale bar: 10 μm. White circles in (a) indicate a few missing domes in the matrix. Yellow circles in (c) highlight the domes damaged because of indentation. (b) Typical approach (black scatters) and retract (red scatters) FDCs, with the former fitted by eq 1 (green line). (d) Stiffness distribution obtained by fitting 54 FDCs with eq 1; red line: histogram cumulative function. (e) Main: distribution of the distance *D* between the feature (2) and the bulk MoS_2_ flake; inset: inner pressure distribution at *D*. (3) Dissipation distribution evaluated by measuring the hysteresis between the approach and retract FDCs (*W*_probe, app_ – *W*_probe, ret_).

We then focused on an array of 8 × 8 openings and pursued a single indentation per dome (loading force of 2 μN). As shown by the AFM image of Figure 3a, 24.5 μm × 24.5 μm in size, 6 domes over 64 (enclosed in white circles) were found damaged prior to performing the indentation procedure. Moreover, a reminiscence of a step in the underneath MoS_2_ flake is imaged from the HSQ-polymer topmost surface, on the right edge of Figure 3a. Figure 3b displays the typical approach (black scatters) and retract (red scatters) FDCs, with the former fitted by eq 1 (green line). As before, apart from the snap-to-contact at the tip–dome contact point, we measure a remarkable pull-in instability, as a second snap-to-contact-like feature at ∼80 nm indentation, followed by a stiff increase of the force (vertical line) when reaching the substrate (bulk MoS_2_), indicated as features (1)–(3), respectively. Again, the distance between (1) and (3) is compatible with the dome’s height, as schematically shown by the cartoon of Figure 1c. Let us first stress that these features, as well as the presence of the pronounced hysteresis between the approach and retract curves, are characteristic of every sampled dome’s FDC. However, as before, the retract curve eventually aligns back to approach FDC to indicate that no permanent damage is induced in the dome. Indeed, Figure 3c shows the AFM morphology acquired on the same area right after the indentations and proves that 95% of the domes have survived the indentation procedure without any damage (only 3 domes over 58, enclosed in yellow circles, were broken by the procedure). Such a result confirms that the peculiarities of the stress–strain diagram, caused by the structural transition discussed before and in Supporting Information 1, are common to all membranes, thus paving the way for the realization of patterned metasurfaces, exhibiting peculiar elastic response to the external stress.^48^

We performed a statistical analysis of 54 reliable FDCs to get some insight into the elastic properties of the domes. Figure 3d shows the histogram of the dome’s stiffness *k*, as derived by fitting each of the approach curves by eq 1 and the histogram cumulative function (in red). The stiffness distribution varies from 12.5 to 21.5 N/m, with a higher number of occurrences between 15 and 20 N/m. Figure 3e shows the distribution of the distance *D* of the pull-in instability (feature (2) and middle panel of Figure 1c) from the bulk flake underneath the membrane. We found that the histogram mainly spreads between 2 and 18 nm, with few occurrences up to 26 nm. These features resemble the ones observed in ref (23) for a suspended graphene membrane attracted via vdW forces to a circular post placed inside a microcavity. We thus correlate the appearance of such instability with the distance at which a vdW interaction sets up between the dome’s membrane and the topmost layer of the underneath bulk flake. Indeed, such an interaction would act as an additional force, having the same direction and orientation as the indenter, and thus, reduce the force needed by the tip to perform indentation. The vdW interaction between the AFM probe and the bulk flake was also separately evaluated by performing indentation on an untreated MoS_2_ crystal; it resulted in a negligible contribution, compared to the membrane-flake MoS_2_ attraction (Supporting Information 2). We can model the unidimensional problem of an indenter pushing against the pressurized dome as a quasistatic process where, point by point, we have
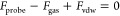
2Here, *F*_probe_ is the force exerted by the AFM probe on the membrane, *F*_gas_ is the force exerted by the gas against the membrane (and against the indentation), and *F*_vdw_ is the vdW interaction force between the dome’s membrane and the topmost layer of the bulk flake. The latter is such that *F*_vdw_ ≠ 0 only at a very small distance (*D* ≲ 26 nm, see Figure 3e). No dependence of FDC’s typical features on the tip speed, in the range 1–900 nm/s, was ever recorded. However, a maximum tip speed of 10 nm/s was employed to guarantee a gradual gas redistribution and membrane rearrangement under the tip apex. The vdW interaction energy acting between the topmost membrane and the bulk flake can be modeled in the geometry of the sphere-plane interaction, as , where *H* and *R*_curv_ are the Hamaker constant and the tip’s curvature radius, respectively, by assuming (i) that in the indented region, the dome’s membrane acquires the same curvature as the AFM probe, and (ii) negligible membrane-flake interaction in the membrane’s region surrounding the indented area, whose distance from the bulk is much higher (Supporting Information 3). By doing so, we can evaluate the vdW force as  for every measured membrane-flake distance *D* (Figure 3e) (here, we used Lifshitz’s approach to calculate the Hamaker constant of an H_2_-mediated interaction between the MoS_2_ monolayer and bulk, *H* = 6.51 × 10^–19^ J; see Supporting Information 3(49)). Once *F*_vdw_ is derived, and *F*_probe_ is known from the FDC curve, we can ultimately derive *F*_gas_ (from eq 2) and *P*_gas_ roughly dividing by the indented area (, assuming π*R*_curv_^2^ as the reference surface). The inset of Figure 3e shows the gas pressure distribution obtained by applying this method, varying in the range of 16–64 MPa. These values are almost one order of magnitude higher than the one evaluated, with a different method and model, in similar size domes.^28^ The reason for the discrepancy is easily explained: in ref (28) the domes were slightly indented and the internal pressure was measured at the dome’s equilibrium size/shape. Here, instead, we measure the gas pressure under deep compression, when the height of the initial topmost point of the dome is indeed reduced from the equilibrium value (90–110 nm) to *D* (see the middle panel of Figure 1c). We compared the results of ref (28) (in terms of the equilibrium pressure *P* and volume *V*), with the pressure measured here, under deep compression, to estimate the change in the dome’s volume under the tip’s action. We found that the pressure in the range of 16–64 MPa is compatible with a volume *V*_compressed_ = (28 ± 9)%*V*_equilibrium_. If we evaluate the volume ideally occupied by a dome, with the same footprint radius as the indented one but with reduced height *D*, herein called *V*_D_, we find *V*_D_ = (30 ± 19)% *V*_compressed_. The missing *V*_compressed_ – *V*_D_ ≈ 70% *V*_compressed_ indicates that besides the reduction in height at the topmost location, a more complex adjustment of the dome’s shape under an indentation occurs. We suggest that the indentation causes a redistribution of the gas, with a consequent formation of a dent at the top of the dome, rather than a uniform decrease of the height at every point of its surface (see the bottom panel of Figure 1c). This result reinforces the assumption that the investigated domes can withstand very large loading and deformations, without being permanently damaged.

Finally, we evaluate the energy dissipated in the indentation process by measuring the hysteresis between the approach and retract FDCs. Indeed, the areas enclosed under the approach and retract curves are a measure of the work done by the tip when pushing against the dome and while being withdrawn, respectively. From eq 2, one can easily derive
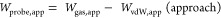
3
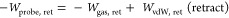
4where the displacement δ⃗, used to evaluate the work *W*, is parallel to *F⃗*_probe_ and *F⃗*_vdW_ and antiparallel to *F⃗*_gas_ during the approach, and vice versa during the retract. The hysteresis (*W*_probe, app_ – *W*_probe, ret_) is thus related to a variation in the work done by the gas and/or by the vdW force during the entire cycle. However, the approach and retract curves involve the same thermodynamical states of the dome (in terms of *P* and *V*), with the final state of the approach being the initial of the retract, and vice versa. Indeed, the dome undergoes a first isotherm transformation from the initial equilibrium thermodynamical state (*P*_eq_, *V*_eq_) to the final (*P*_compressed_, *V*_compressed_) (approach/loading) and a second isotherm transformation (same temperature as before) from the initial thermodynamical state (*P*_compressed_, *V*_compressed_) to the final (*P*_eq_, *V*_eq_) (retract/unloading). Thus, *W*_gas_ is expected to be the same, in modulus, for approach and retract (*W*_gas,app_ = *W*_gas,ret_), but with opposite sign, as representative of compression during the approach and expansion during the retract. Therefore, by adding up eqs 3 and 4, we get
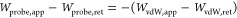
5thus establishing the equivalence, in modulus, between the total work done by the probe (which corresponds to the total energy dissipated during a loading/unloading cycle and is ultimately equal to the area of the measured hysteresis loop) and the total work done by the vdW force, against the gas, to favor the re-adhesion between the dome’s membrane and the topmost layer of the bulk flake. Figure 3f displays the measured dissipated energy distribution, with values mostly ranging between 0.5 and 2.3 × 10^–14^ J and only a few occurrences at higher dissipation. By roughly normalizing these values to the area under the tip apex (π*R*_curv_^2^), we find (4.7 ± 2.5) × 10^–21^ J/Å^2^ = 30 ± 16 meV/Å^2^, remarkably close to the adhesion energy measured in refs (28) and (50) with different approaches, as well as to the one found for different 2D materials and their substrates.^1^

We underline that besides the features discussed and shown by the FDC of Figure 3b, always present in any of the large-indentation FDCs, examples and details of a few curves disclosing a more complicated behavior under the indentation are given in Supporting Information 4.

**Figure 4:**
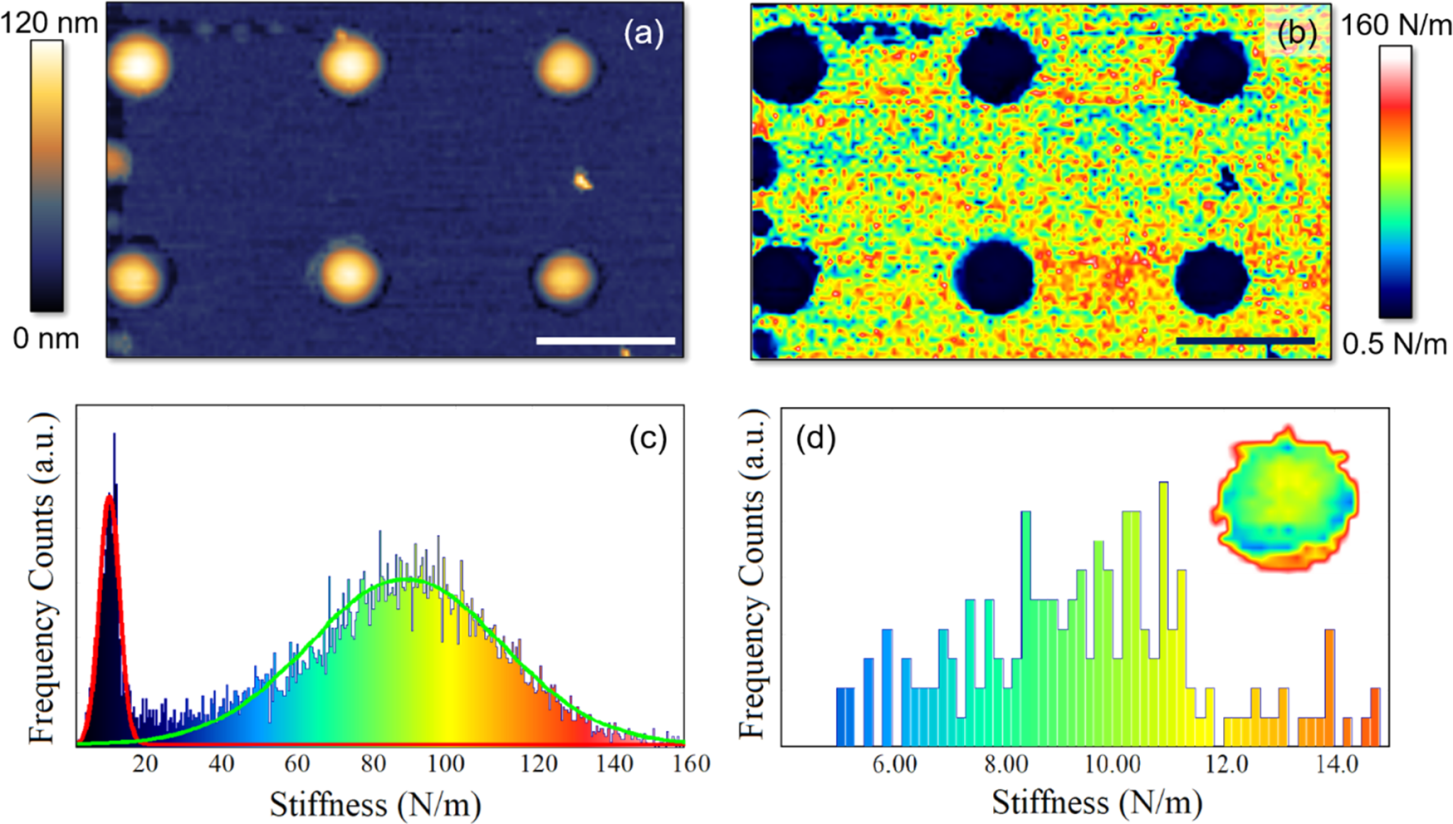
(a, b) Topography and stiffness maps as obtained by performing QI-AFM; scale bar: 2 μm. (c) Stiffness distribution as extracted from (b). Red and green curves are Gaussian fits centered at 8 ± 5 and 86 ± 51 N/m, respectively. (d) Main: stiffness distribution obtained by including only the dome’s area; inset: stiffness map of a single dome.

In addition, we performed quantitative-imaging AFM (QI-AFM) measurements obtained by acquiring an FDC per pixel of the selected scan area. From the measured FDC, one can reconstruct the topography by mapping the spatial variation of the tip/surface contact point (dependent on surface protrusions) and the stiffness map by deriving *k* from the fit of each curve. We performed QI-AFM by using a loading setpoint of 120 nN, and the outcoming morphology and stiffness maps, 8.3 μm × 5.1 μm in lateral size, are shown in Figure 4a,b, respectively. A clear one-to-one correspondence between the morphological and elastomechanical properties of the scanned area is found, with the domes being softer than the surrounding HSQ mask. Figure 4c shows the stiffness distribution as extracted from Figure 4b: two well-distinct peaks appear, both fitted by a gaussian distribution. The softer peak, fitted by the red curve, is representative of the dome’s mechanical response and is centered at 8 ± 5 N/m, whereas the hardest peak, fitted by the green curve and representative of the mask’s response, is centered at 86 ± 50 N/m. By employing a Hertz fit of FDCs acquired in the mask’s region, we found the HSQ Young’s modulus ranging from 2 to 12 GPa, consistent with ref (51). The softer peak, at 8 ± 5 N/m, describes instead the mechanical response of the domes, across their whole surface, and thus includes the stiffness behavior at the topmost locations as well as that along the edges. In this framework, Figure 4d details the mechanical response of the dome as a function of the indenter position. A zoom of the stiffness map, centered at the dome’s location, is displayed as inset. One can notice that the stiffness measured on the topmost region of the domes (in yellow, ∼12 N/m) is higher than that at the edges, gradually decreasing from the top toward the bottom (moving from green to blue contrast). A softening of the mechanical response of the dome is indeed fully expected when pushing far away from the center. In fact, by doing so, it is easier to induce a redistribution of the internal gas, compared to the case of indenting at the topmost location since the gas volume under the tip apex is increasingly smaller as the indenter moves further away from the center. Finally, a red contrast (highest stiffness) is measured at the border between the dome and the opening’s edge, where the tip simultaneously feels the action of the MoS_2_ membrane of the MoS_2_ flake and of the HSQ mask.

**Figure 5:**
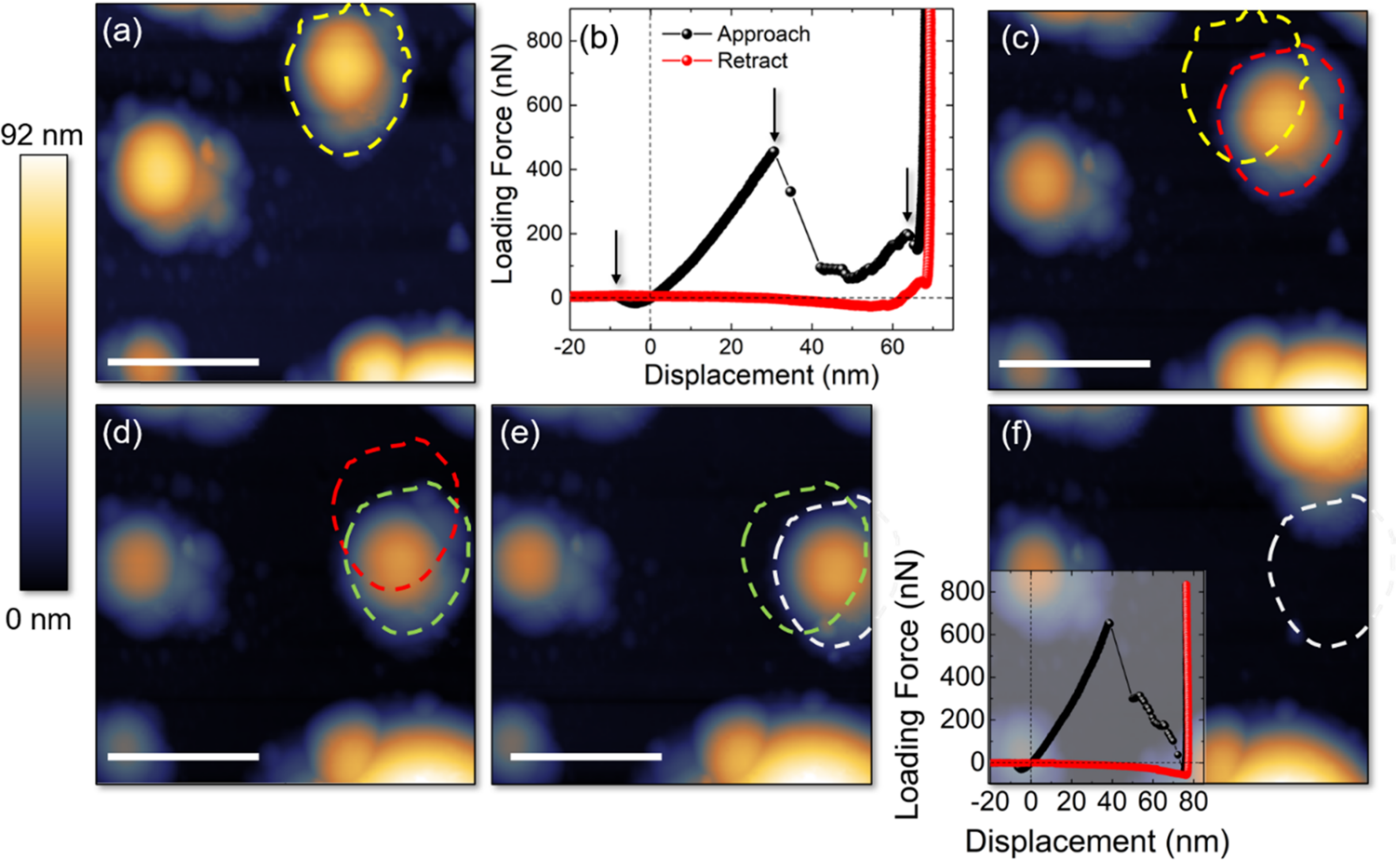
(a, c, d–f) AFM topography, 2.5 μm × 2.5 μm in lateral size, of the same scan area before and after indenting the highlighted dome multiple times. Dashed lines indicate the original position of the dome and its shift after the indentation; scale bar: 1 μm. (b) Typical FDC obtained when indenting on the unconstrained dome. (f) Inset: FDC measured before dome’s merging.

Finally, we explored the behavior of unconstrained domes subjected to deep indentation. Figure 5a shows a tapping-mode AFM image, 2.5 μm × 2.5 μm in lateral size, of an uncoated MoS_2_ flake surface, revealing the nucleation of spontaneous domes of random size and in random locations. As one can notice, these domes are slightly less spherical in shape, compared to the constrained ones, and are affected by the satellite smaller bubbles along their perimeter. We focused on the yellow-dashed dome and performed a loading procedure by exerting a maximum force of 2 μN. The corresponding FDC is shown in Figure 5b. This time, the approach curve (in black) shows the appearance of three snap-to-contact-like features (indicated by black arrows): the first at the tip–dome contact point, the second at ∼30 nm indentation, and the third ∼3 nm away from the bulk flake. The position of the second snap-to-contact allows us to exclude the vdW membrane-flake interaction as a possible source of the phenomenon, which is instead attributed to the feature occurring ∼3 nm away from the flake. Indeed, the distance of the second snap-to-contact from the bulk, ∼40 nm, is higher than the expected vdW range (*D* ≲ 20 nm). In addition, the retract FDC (red scatters) does not overlap any longer with the approach one, as to indicate a change in the indented object. Indeed, Figure 5c shows the AFM topography of the same scan area after indentation: the yellow highlighted region indicates the original position of the dome, which is now down- and right-shifted (the new position is enclosed in the red dashed line). We performed three more cycles of subsequent indentation and imaging, exerting a maximum force of 2 μN, and found a significant shift of the dome’s position after each process. Figure 5d,e details the dome’s movement from the red to the green region (d) and from the green to the white region (f). In the last step, we measured a much more drastic change in the dome: the FDC displayed in Figure 5f shows that, this time, the force not only abruptly decreases in correspondence of the second snap-to-contact but it also reaches negative values, as to indicate a dramatic change in the tip–sample interaction. The AFM map performed afterward (Figure 5f) shows that the dome has moved considerably, eventually merging into a bigger one on the top-right corner of the image. We then evaluated the energy dissipated in each step (*W*_probe,app_–*W*_probe,ret_), as the energy cost of partially delaminating the MoS_2_ flake (to allow the dome movements) under the action of the gas, compressed by the indentation. The obtained values are reported in the second column of Table 1. While the energy dissipated during the dome’s movement from Figure 5a to c, from c to d, and from d to e are very close to each other, the energy dissipated during the last step (from Figure 5e to f) is, as expected, higher (3.10 × 10^–14^ J to be compared to 1.21–1.88 × 10^–14^ J). To get some insight into the energy density dissipated during the process, we performed point-by-point subtractions of the AFM maps, before and after each indentation, and evaluated the area involved in the delamination, as a consequence of dome’s movements. The results are listed in the third column of Table 1. Here, the delaminated areas of cycles #3 and #4 are representative of a lower limit estimate since the dome has partially moved outside of the imaged area. The corresponding energy densities are reported in the fourth column of Table 1 and vary between 3.68 and 7.80 meV/Å^2^, remarkably close to the values measured in refs (28) and (53) in randomly formed unconstrained domes. While it would be tempting to associate these values to the adhesion energy, it is to be noted that (i) these values are remarkably smaller than those found in ordered domes,^28,50^ which (ii) are also in better agreement with theoretical estimates (∼20 meV/Å^2^ for MoS_2_).^50,52^ Indeed, indentations performed on ordered domes—for which the dome’s position remains unchanged, and the AFM tip stays aligned with the dome’s summit—are probably better suited for determining the *adhesion energy*, even though possibly slightly affected by the surrounding constraint.^28^ On the other hand, the values found for unconstrained domes should likely be regarded as estimates of the energy required to *move* a dome across the flake’s surface.

**Table 1:** Dissipated Energy, Delaminated Area, and Dissipated Energy Density for Each Cycle of Indentation Reported in Figure 5

cycle #	energy (J × 10^–14^)	delaminated area (Å^2^ × 10^6^)	energy density (meV/Å^2^)
1 (Figure 5f (a–c))	1.45	24.6	3.68
2 (Figure 5f (c–d))	1.88	28.6	4.10
3 (Figure 5f (d–e))	1.21	≥17.3	≤4.02
4 (Figure 5f (e–f))	3.10	≥24.7	≤7.80

## Conclusions

We used AFM-based nanoindentation to investigate the elasticity, hardness, and adhesion of microsized H_2_-filled MoS_2_ domes, whose nucleation position and dimension were controlled by lithographic masks opaque to hydrogen.^28,29^ We showed that the engineered domes represent an excellent platform to derive information on the membrane elasticity and vdW interlayer interactions, as well as to develop a new methodology for studying the loading force curves describing indentation against pressurized objects. Remarkably, we demonstrated that when the domes are constrained in their nucleation position by the mask, they exhibit exceptionally high robustness to the external loading. Indeed, 95% of the sampled domes reacted to the large AFM load or crushing procedure (indentation depth as large as the whole dome’s height), with a full recovery of the dome’s shape and size, upon unloading. Moreover, they exhibited a peculiar force–displacement diagram, made of two elastic branches, separated by a big hysteretic region, accessible when performing a whole loading–unloading cycle. Such a mechanical response resembles the one of conventional, superelastic, alloys.^46^ A quantitative analysis of the force–displacement curves and the related hysteresis allowed us to derive the interlayer adhesion energy equal to 30 ± 16 meV/Å^2^. Unconstrained domes, on the other hand, showed a lateral slippage, under the large AFM indentation, with a consequent local MoS_2_–MoS_2_ delamination/adhesion. In this case, the indentation is far from being reversible since the dome’s position is modified by loading. This circumstance allowed us to derive a delamination energy density associated with the dome movement of about 6 meV/Å^2^, in agreement with previous results.^28,53^ Our findings, on the one hand, establish the important role of the superimposed constraint into the overall mechanoelastic properties of these blisters; on the other hand, open the way for deterministic handling of the domes. In fact, the possibility to precisely position a highly strained feature—not unlike those that have consistently shown the ability to emit single photons^54−56^—would represent a significant breakthrough in 2D materials research. Engineering the dome’s movements to a fully controllable level, with nanometer-scale precision, could indeed have profound consequences on the quantum photonic applications of 2D materials.^57^ In addition, the capability to induce a controlled merging of several domes may result in the creation of strained regions with the desired surface area.

## Methods

### Sample Preparation and Proton Irradiation

Thick MoS_2_ flakes were first mechanically exfoliated from commercial MoS_2_ crystals (from 2D semiconductors) by scotch tape, in such a way that a part of the crystal remained on the tape. By making the tape adhere to the SiO_2_/Si substrate and slowly peeling the tape off, several flakes with a thickness of hundreds of layers were left on the substrate. The whole process was done in air and at ambient conditions. A part of the substrate was then coated with hydrogen silesquioxane masks and subjected to electron beam lithography, as detailed in the next section. With this procedure, both patterned and unpatterned flakes were present on the same sample. The samples were subsequently ion-irradiated with a Kaufman source.^24^ To perform irradiation, the sample was mounted on a metallic holder so as to be grounded. The holder was placed in a vacuum chamber, which was brought to a base pressure of <1 × 10^–6^ mbar, and the temperature was increased to a value in the range of 120–150 °C. Hydrogen ions were obtained in an ionization chamber and accelerated by a system of grids, thus irradiating the sample with an ion beam with energy in the range of 10–20 eV. The samples were irradiated with a total dose in the range of 6–7 × 10^16^ ions/cm^2^.

### Electron-Beam Lithography Patterning

The engineered formation of MoS_2_ domes was achieved via the fabrication of H-opaque masks, performed by means of electron-beam lithography (EBL, Vistec EPBG 5HR system working at 100 kV). The engineering procedure is as follows:^24^ a hydrogen silesquioxane (HSQ) negative-tone e-beam resist is spun onto the sample surface. EBL is then performed to get octagonal openings of predetermined dimensions and with the desired ordering on the HSQ layer. An electron dose of 300 μC/cm^2^ and an aqueous development solution of tetramethyl ammonium hydroxide at 2.4% were used for the patterning of the HSQ masks. To make the resist act as a constraint during the dome formation process, a resist thickness of 30–50 nm was employed. Moreover, the HSQ being a negative-tone resist, only the area irradiated with the electron beam is subjected to an internal modification and, consequently, only the electron-irradiated area remains on the sample upon HSQ development.

### Atomic Force Microscopy Measurements

AFM images were acquired using a JPK Nanowizard III, equipped with Vortex electronics, in the standard tapping mode technique using an LTESP Si probe (from Bruker). The elastic properties were measured by exerting a maximum loading force as high as 2 μN, at the center of the pressurized membrane, to perform local nanoindentation AFM experiments. The indentation depth δ is determined as δ = Δ*z*_piezo_ – Δ*z*_tip_, where Δ*z*_piezo_ is the displacement of the AFM piezotube and Δ*z*_tip_ is the deflection of the cantilever, measured by the photodiode. To preserve the tip’s shape and size, cantilever characterization was carried out, prior to performing the indentation, by employing the contact-free method,^58^ which does not require preceding force–distance curve acquisition on a hard material to determine cantilever sensitivity, although it only applies to rectangular cantilevers. The knowledge of the cantilever’s geometrical dimensions (length and width) as well as the physical properties of the environment/medium (density and viscosity), where the measurements are performed, is mandatory to derive reliable values of the spring constant *s* and deflection sensitivity δ_c_, besides resonance frequency *f*_0_ and quality factor, by employing thermal noise measurements. The presented experiments were performed at room temperature under ambient conditions (density: ≈1.185 kg/m^3^ and viscosity: ≈18.37 μPa × s) and by using rectangular cantilevers of 225 μm in length and 35 μm in width, having, on average, δ_c_ ≈ 40 nm/V and *s* ≈ 50 N/m. δ_c_ and *s* were tested afterward by employing the standard contact-based method at the end of each measurement run on a hard substrate (e.g., Si/SiO_2_), confirming the results of the contact-free procedure.

All of the data were analyzed using WsXM, Scanning Probe Image Processor (SPIP), Origin, and Mathematica. The measured “vertical-deflection vs piezo-movement” curves were systematically converted into the “force vs distance” ones, by computing the cantilever contribution to the total deflection measured and by subtracting it to isolate only the sample response to the externally applied load.

## Supporting Information Available

Conventional superelasticity vs the elasticity of constrained MoS_2_ domes; AFM nanoindentation of the MoS_2_ bulk crystal; van der Waals potential and force simulation for H_2_-mediated MoS_2_–MoS_2_ interaction; and force–displacement curves as a probe of dome’s inner structure (PDF)
