# Supplementary material for: Exceptional Elasticity of Microscale Constrained MoS2 Domes
Source: ACS Appl Mater Interfaces. 2021 Oct 1;13(40):48228–38. doi: 10.1021/acsami.1c13293 (PMC8517950; doi:10.1021/acsami.1c13293)
Supplement: Supplementary file 1 — am1c13293_si_001.pdf [file am1c13293_si_001.pdf]

Supporting Information: Exceptional elasticity of micro-scale constrained MoS_2 domes

*Cinzia Di Giorgio[1,2], Elena Blundo[3], Giorgio Pettinari[4], Marco Felici[3], Antonio Polimeni[3], and Fabrizio Bobba[1,2,5]**

1. Department of Physics E.R. Caianiello, University of Salerno, 84084 Fisciano, Italy
2. INFN, Sezione di Napoli, Gruppo Collegato di Salerno, Complesso Universitario di Monte S. Angelo, 80126 Napoli, Italy
3. Physics Department, Sapienza University of Rome, 00185 Rome, Italy
4. Institute for Photonics and Nanotechnologies (CNR-IFN), National Research Council, 00156 Rome, Italy
5. CNR-SPIN, 84084 Fisciano (SA), Italy

KEYWORDS. Elasticity; Two-dimensional materials; bulged membranes; nano-indentation; force-distance curves; adhesion energy.

1. Conventional superelasticity vs the elasticity of constrained MoS_2 domes

The phenomenon of superelasticity (or pseudoelasticity) in conventional shape-memory alloys is characterized by i) a fully reversible response to an applied stress; and ii) a peculiar shape of the stress-strain (σ-ε) diagram, as the one representatively shown in Figure S1(a) (extracted from [1]). The latter exhibits two subsequent linear elastic branches (O-A and B-C) separated by a hysteresis (ABDE), occurring because of a reversible structural transformation (from the austenitic, O-A, to the martensitic, B-C, phase) of the stressed crystal [1]. For sake of comparison, Figure S1(b) shows a typical force-displacement curve (FDC) resulting from the indentation of constrained MoS_2 domes.

These latter react to the performed crushing procedure (indentation as large as the dome's height) with i) a full reversibility of the loading/unloading process (approach, and retract curves - black and red, respectively - overlap upon releasing the loading); ii) a stress-strain diagram qualitatively similar to the one of conventional superelastic materials - in our experiments, the stress σ is applied through the loading force exerted by the tip, and the strain ε is proportional to the distance travelled inside the dome (or displacement). Importantly, the similarity in shape (the two elastic branches separated by a large hysteresis) is driven by a similar underlying phenomenology: both in the case of conventional superelastic materials and engineered MoS_2 domes, the hysteresis is driven by a structural transition, between the two different phases of the crystal in the first, and between the two different states of the system (suspended MoS_2 monolayer, and bulk constituted by the MoS_2 monolayer stuck on the parent substrate) in the second. The two linear branches are then the elastic response of the two crystalline phases in conventional superelastic materials, and that of the bulged MoS_2 monolayer and of the bulk material in our system.

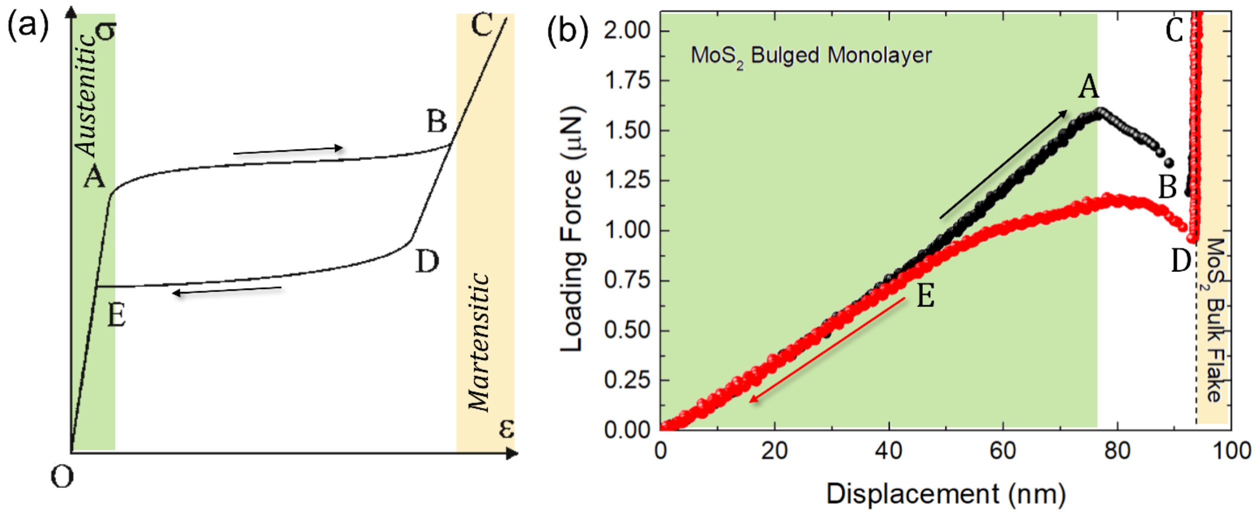

Figure S1. (a) typical stress-strain (σ-ε) diagram of a conventional shape-memory alloy, as extracted from [1]; (b) typical FDC resulting from the indentation of a constrained MoS_2 dome.

2. AFM Nanoindentation of MoS_2 bulk crystal

To discriminate the contribution of the van der Waals (vdW) attraction between the AFM probe and the bulk MoS_2, when performing large-range indentations on domes, we pursued atomic force spectroscopy (AFS) of an untreated MoS_2 crystal. Figure S2 shows the comparison between loading and unloading force vs displacement curve (FDC) as acquired on both bulk and membrane MoS_2, by using the same AFM probe. We used LTESP Si tips, from Bruker, having in average a resonance frequency of 175 kHz, a deflection sensitivity of 40 nm/V and a spring constant of about 50 N/m. As expected, no additional pull-in instabilities are ever measured when indenting on the bulk, besides the snap-to-contact at the tip-MoS_2 contact point, due to the vdW attraction between the AFM probe and the crystal's surface. Noteworthy, the jump-to-contact force in this case varies in between 10-20 nN, more than one order of magnitude smaller than the second snap-to-contact (feature (2)) appearing when indenting on a dome, thus making the AFM probe-bulk MoS_2 interaction negligible with respect to the membrane-bulk MoS_2 counterpart. Interestingly, as shown in the inset of Figure S2, also the shape of the first snap-to-contact (feature (1)) appears much different between bulk crystal and dome, the latter exhibiting a much slower and smoother force variation when AFM probe and membrane get closer and closer to each other. Such a finding demonstrates the strong reciprocal interaction between the tip and the membrane, with the membrane getting attracted and physically moving toward the probe as soon as the vdW forces set up (10-20 nm distance). In addition to this, we evaluated the hysteresis (if any) between approach and retract FDCs, when indenting on MoS_2 crystal, resulting in ~$0.04\text{-}0.1\times10^{-14}$ J – more than one order of magnitude smaller than that found when indenting on domes (Figure 3f – main text). Obviously, we underline that the indentation on the crystal gets vertical much earlier compared to the domes, being i) the bulk much harder and less prone to deformation, and ii) the used cantilever proper to match and measure the elastic properties of the domes rather than that of the flake.

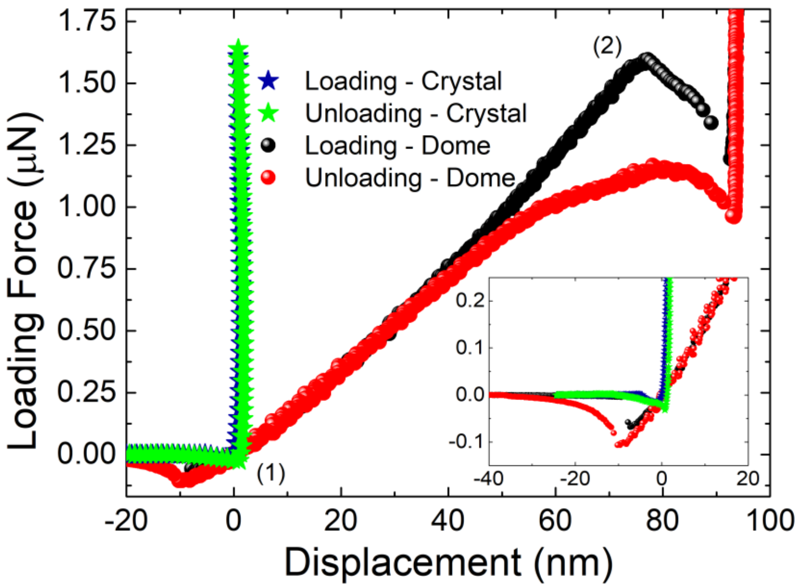

Figure S2. Main: typical FDCs acquired on untreated MoS_2 crystal (approach and retract cruves are blue and green stars, respectively) and on the domes (approach and retract are black and red spheres, respectively). Inset: zoom on snap-to-contact (1).

3. Van der Waals potential and force simulation for H_2-mediated MoS_2-MoS_2 interaction

Figure S3 plots simulated vdW potential and force curves for a sphere-surface modelled interaction, $U = -\frac{HR_{curv}}{6\pi D}$ and $F_{vdw} = -\frac{dU}{dD} = -\frac{HR_{curv}}{6\pi D^2}$, respectively [2]. Here R_{curv} is the radius of the indenting AFM-probe, ideally assuming that the indented membrane acquires the same curvature, D is the distance between top-most membrane and underneath bulk flake, and H is the Hamaker constant. We used the Lifshitz theory to evaluate the Hamaker constant for a top-most MoS_2 membrane interacting with the bulk MoS_2, over a third medium (H_2) [2]:

$$H = \frac{3}{2}k_B T\left(\frac{\varepsilon_1 - \varepsilon_3}{\varepsilon_1 + \varepsilon_3}\right)\left(\frac{\varepsilon_2 - \varepsilon_3}{\varepsilon_2 + \varepsilon_3}\right) + \frac{3hf_e}{8\sqrt{2}}\frac{(n_1^2 - n_3^2)}{\sqrt{n_1^2 + n_3^2}}\frac{(n_2^2 - n_3^2)}{\sqrt{n_2^2 + n_3^2}}\frac{1}{(\sqrt{n_1^2 + n_3^2} + \sqrt{n_2^2 + n_3^2})} = 6.51 \times 10^{-19} J$$

The indexes 1,2, and 3 refer to monolayer MoS_2, bulk MoS_2 flake and H_2, respectively. Moreover, ε_i, h, f_e and n_i are static dielectric constant, Plank constant, plasma frequency, and refractive index in the visible range, respectively. By using the values reported in Table S1, we found $H = 6.51 \times$

$10^{-19} J$, slightly higher than the MoS_2-MoS_2 interaction energy in vacuum (or inert air) of 1.25 - 2.21 × $10^{-19} J$ [3].

As shown by Figure S3, the vdW interaction strongly decays in the first 10 nm distance and becomes totally negligible at 20 nm and higher.

	ε_i	n_i	f_e
MoS_2-ML	~6.2[4]	3.8256 (@ ~580nm) [7]	
MoS_2-bulk	~6.8 [4]	4.7739 (@ ~580nm) [7]	2.12×10^{15} [2]
H_2	1.0002538 – 1.2[5,6]	1.00013881 (@ ~580nm) [8]	

Table S1. Values of static dielectric constants (ε_i), refractive indexes (n_i) and plasma frequency (f_e) used to evaluate the Hamaker constant.

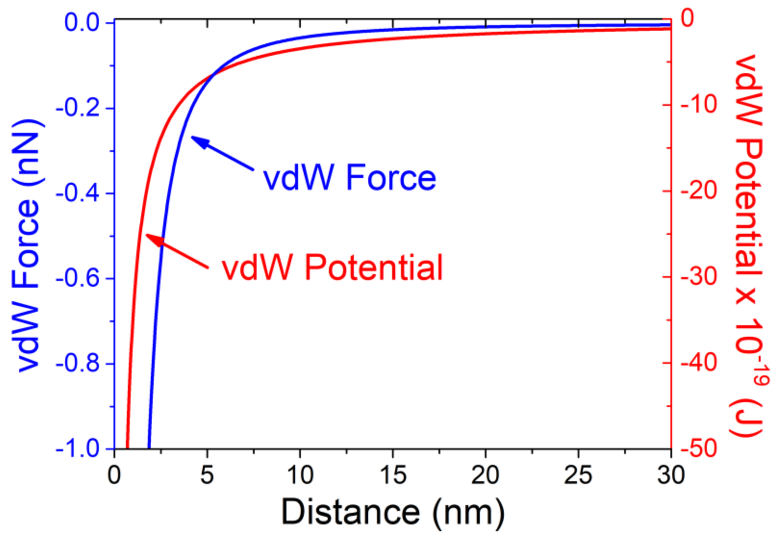

Figure S3. Simulated vdW potential (red) and force (blue) curves for a monolayer MoS_2 interacting with bulk MoS_2, over H_2 medium.

4. Force-displacement curves as probe of dome's inner structure

Figure S4(a) and (b) show two different approach curves affected by a change in slope, happening at ~70 nm and ~50 nm indentation, respectively (gray highlighted areas). We correlate these occurrences to the presence of a second smaller dome, fully enclosed into the outer one (the latter being the only one accessible when performing AFM surface topography). Indeed, by considering this, the change in slope, highlighted in gray, can be attributed to the reciprocal attraction between the membranes of the outer and inner domes, being attracted one to the other, in downward and upward direction, respectively. Indications on the sporadic existence of inner domes are given, sometimes, after breaking the top-most membrane. As an example, figure S4(c) shows an AFM image, 1.4 μm × 1.4 μm in size, where the bottom of the opening is exposed because of dome breaking. The original dome's membrane, broken and folded on itself, is recognizable and indicated by a white arrow, together with two smaller domes lying on the bulk flake, underneath the membrane.

We then fitted the FDCs of figure S4(a) and (b) by applying the model of equation (1)-main text, two times per each curve, herein called Fit#1 and Fit#2 for the outer and the inner dome, respectively. Depending on the distance travelled inside the outer or the inner dome, the fit can be dominated by the linear or by the non-linear component. For instance, the first branch of figure S4(a), corresponding to the outer dome, displays the combination of both linear and non-linear behavior (Fit#1, in green, with a transition at 5.3 nm and stiffness of 14 N/m), whereas the second branch, corresponding to the inner dome, is only dominated by the linear component (Fit#2, in red), since it is only ~10 nm far from the second snap-to-contact. On the contrary, the outer dome of figure S4(b), whose behavior is mainly represented by the first branch of the FDC, shows mostly a linear trend (Fit#1, in green, stiffness is 14.5N/m and the transition is at 23nm), whereas the inner dome (second branch of FDC) can be fitted by summing up both linear and non-linear behavior (Fit#2, in red). Indeed, in this case, the inner dome's membrane is ~25 nm far from the second snap-to-contact. Whether the inner dome is taller in the second case, or it is not centered in the first one is hard to say without damaging

irreversibly the outer membrane. Moreover, the stiffness measured in the inner domes is 39 N/m and 27.8 N/m for FDC S4(a) and S4(b) respectively. These higher stiffness values can be attributed to (1) a convolution of inner and outer dome's mechanical properties and (2) the smaller size of the inner dome. Indeed, the inner pressure has been demonstrated to increase as the dome's size decreases [9-11], thus affecting the stiffness. On the other hand, the presence itself of a bulged dome, enclosed into another one, is a confirmation that $P_{inner} > P_{outer} > P_{environment}$. A proper deconvolution of these effects would necessarily require the exact knowledge of inner dome's size, shape, and centering. Finally, Figure S4(d) shows the FDC acquired on the top of the broken and folded membrane (indicated by the white arrow in figure S4(c)). Also in this case, the approach FDC (black scatter) shows two subsequent snaps-to-contact, at the tip-membrane contact point, and when the membrane, being pushed downward by the tip's action, reaches the bulk MoS_2 flake. This time, besides the hysteresis between approach and retract curves, one can notice that the indentation has given rise to an irreversible phenomenon: the retract FDC does not overlap back to the approach one at any distance, indicating that the adhesion between the membrane and the flake has dominated the process, keeping the two layers together even after the tip has been pulled away, at the cost of 0.9 x 10^{-15} J (1.8 meV/A^2). This result points toward the big role of the gas in establishing back the shape of the domes, after the indentation procedure. Indeed, during the retract, the inner pressure competes with the adhesion energy and eventually wins, making the indentation processes on the domes fully reversible.

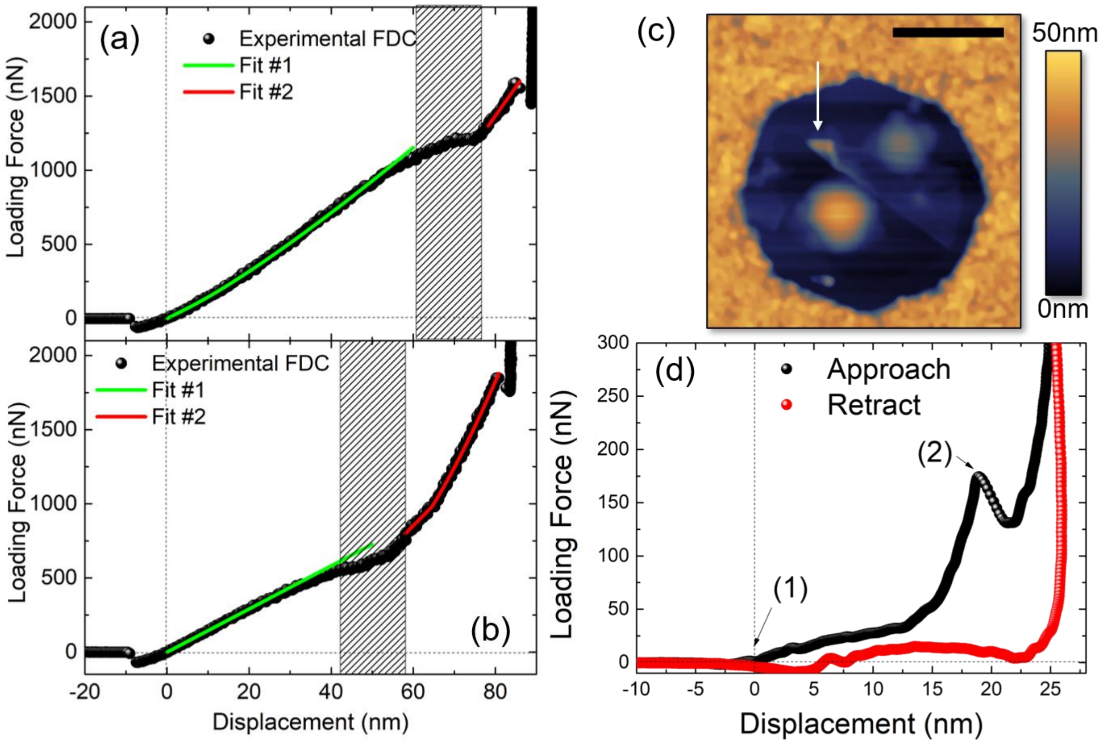

Figure S4. (a)-(b): Two examples of approach FDCs showing an abrupt change in slope at ~70 nm (a) and ~50 nm (b), highlighted in gray. The curves have been fitted by implying equation (1)-main text twice per each curve: Fit#1, in green, and Fit#2 in red describe the behavior of the first and second branch, before and after the change in slope, respectively. (c) Tapping-mode AFM topography, 1.4 µm × 1.4 µm in lateral size, performed after dome's breaking. Scale bar is 0.5 µm; (d) approach (black scatters) and retract (red scatters) FDCs acquired on top of the membrane, at the location pointed by the white arrow in (c).

REFERENCES

[1] Bhattacharya, K.; Schlömerkemper, A. Stress-Induced Phase Transformations in Shape-Memory Polycrystals. *Arch. Rational Mech. Anal.* **2010**, 196, 715.

[2] Leite, F.L.; Bueno, C.C.; Da Róz, A.L.; Ziemath, E.C.; Oliveira Jr., O.N. Theoretical Models for Surface Forces and Adhesion and Their Measurement Using Atomic Force Microscopy. *Int. J. Mol. Sci.* **2012**, 13, 12773.

[3] Krajina, B.A.; Kocherlakota, L.S.; Overney, R. M. Direct Determination of the Local Hamaker Constant of Inorganic Surfaces based on Scanning Force Microscopy. *J. Chem. Phys.* **2014**, 141, 164707.

[4] Laturia, A.; Van de Put, M.L.; Vandenberghe, W.G. Dielectric Properties of Hexagonal Boron Nitride and Transition Metal Dichalcogenides: from Monolayer to Bulk. *npj 2D Materials and Applications* **2018,** 2, 6.

[5] Lide, D.R. Handbook of Chemistry and Physics 78th Edition. *CRC Press* **1997**.

[6] DK-Wert-Liste_DE-EN-FR.xls (vega.com)

[7] Refractive index of MoS2 (Molybdenum disulfide) – Zhang

[8] Refractive index of H2 (Hydrogen) - Peck

[9] Khestanova, E.; Guinea, F.; Fumagalli, L.; Geim, A. K.; Grigorieva, I. V. Universal Shape and Pressure inside Bubbles appearing in van der Waals Heterostructures. *Nat. Commun.* **2016**, 7, 12587.

[10] Di Giorgio, C.; Blundo, E.; Pettinari, G.; Felici, M.; Lu, Y.; Cucolo, A.M.; Polimeni, A.; Bobba, F. Nanoscale Measurements of Elastic Properties and Hydrostatic Pressure in H_2-Bulged MoS_2 Membranes. *Adv. Mater. Interfaces* **2020**, 7, 2001024.

[11] Blundo, E.; Yildirim, T.; Pettinari, G.; Polimeni, A. Experimental Adhesion Energy in van der Waals Crystals and Heterostructures from Atomically-Thin Bubbles. *Phys. Rev. Lett.* **2021**, 127, 046101-1.
